# The effect of COVID-19 on the frequency of *Pneumocystis jirovecii* pneumonia: a monocentric, retrospective, and observational study

**DOI:** 10.1186/s12879-023-08545-w

**Published:** 2023-10-17

**Authors:** Valentina Del Prete, Giovangiacinto Paterno, Oreste Cennamo, Federica Berrilli, David Di Cave

**Affiliations:** 1 Department of Clinical Sciences and Translational Medicine, University of Rome Tor Vergata, Rome, Italy; 2 Department of Biomedicine and Prevention, University of Rome Tor Vergata, Rome, Italy

**Keywords:** *Pnemocystis Jirovecii* pneumonia, COVID-19, Bronchoalveolar lavage, Genotypic analysis

## Abstract

**Background:**

*Pneumocystis jirovecii* pneumonia (PCP) and SARS-CoV2 share some similarities in their effects on the respiratory system, clinical presentation, and management. The COVID-19 pandemic required rapid action to curb transmission and mitigate its lethiferous impact. Non-pharmaceutical interventions (NPIs) were globally adopted. We hypothesized that these measures reduced the transmission and acquisition of *P. jirovecii* in both hospital and community settings.

**Methods:**

We conducted a retrospective observational study on 2950 respiratory specimens from patients with suspected pulmonary infection, analyzed at the Laboratory of Parasitology Unit of the Policlinico Tor Vergata of Rome, Italy, from January 2014 to December 2022.

**Results:**

We show a significant reduction in the frequency of PCP in the COVID-19 pandemic era compared to the previous period. Among the four sequence types of *P. jirovecii* identified, genotype 1 was the most prevalent (37%). We observed a non-significant trend of decreasing cases with genotype 1 and increasing cases with genotype 3 over the study period.

**Conclusions:**

The nationwide implementation of NPIs against COVID-19 may have changed the microbiological landscape of exposure, thereby decreasing the exposure to *P. jirovecii* and consequently reducing the incidence of PCP.

## Introduction

*Pneumocystis jirovecii* pneumonia (PCP) and 2019-coronavirus disease (COVID-19) are two respiratory diseases that have significant clinical implications. Although they are caused by different infectious agents, they share certain similarities in terms of their impact on the respiratory system, clinical presentation, and management.

*P. jirovecii* is an opportunistic ascomycetous fungus that causes a life-threatening interstitial pneumonia in immunocompromised patients [1]. This infectious agent has a specific tropism for the cells of the pulmonary epithelium, attacks the alveoli and the extracellular spaces, and is able to sustain itself thanks to the alveolar fluids [2]. A range of supporting evidence indicates that *P. jirovecii* can be transmitted from person to person, possibly via aerosols. Additionally, *P. jirovecii* can also be carried asymptomatically (colonization) by individuals in the general population. [3–5] The environmental reservoir and survival of *P. jirovecii* outside the human host are still unclear. Diagnosis of PCP is typically made by a combination of clinical suspicion, radiographic findings, and laboratory testing, including bronchoscopy with bronchoalveolar lavage [6]. The risk factors for PCP include HIV infection, hematological malignancies, solid organ transplantation, autoimmune diseases, and use of immunosuppressive drugs. [7–9]

The World Health Organization declared the outbreak of COVID-19 a global pandemic on 11 March 2020 [10]. COVID-19 primarily affects the respiratory system, causing symptoms such as fever, cough, fatigue, and shortness of breath. In severe cases, COVID-19 can lead to acute respiratory distress syndrome (ARDS), sepsis, and multi-organ failure. The rapid spread and high fatality rate associated with COVID-19 demanded rapid action to curb the transmission and mitigate the pandemic’s impact. In response, policymakers have instituted globally an array of non-pharmaceutical interventions (NPIs) such as social distancing, facial masking, hand washing, travel restriction, self-isolation, and quarantine to mitigate the spread of the virus and ease the burden on healthcare systems [11].

Based on this recent substantial proof of *P. jirovecii* as a transmissible fungus, we hypothesized that the multifarious infection control measures and their strong compliance during the COVID-19 pandemic possibly led to prevent the intra-hospital transmission and community acquisition of *P. jirovecii*. Therefore, we investigated the change in *P.* jirovecii detection in the COVID-19 pandemic era to evaluate the effect of infection control measures.

## Materials and methods

We performed a retrospective observational study involving 2950 respiratory specimens (bronchoalveolar lavage, BAL, and tracheobronchial aspirate fluid) from immunocompetent and immunocompromised patients with suspected pulmonary infection, consecutively analyzed at the Laboratory of Parasitology of the Polyclinic Tor Vergata in Rome, Italy from January 2014 to December 2022. All these specimens were collected for the detection of *P. jirovecii*, as requested by the physician. We excluded all repeated results for *P.* jirovecii tests in each patient. The pre-COVID-19 and post-COVID-19 periods were defined as before and after 1 January 2020. Samples from the same patient, even if collected at different times, were excluded from the analysis.

For all samples, the detection of *P.* jirovecii was based on immunofluorescent procedures (DFA) with monoclonal antibodies (MERIFLUOR(r) Pneumocystis, Meridian Diagnostic, Cincinnati, OH, USA) [12, 13] and on nested-PCR. DNA extraction was performed from biological samples using a commercially available kit (QIAamp DNA kit; QIAGEN, Hilden, Germany) according to the manufacturer’s instructions. The nested PCR of mtLSU-rRNA gene (260-bp fragment length) was performed by using the primers pAZ102-H and pAZ102-E for the first amplification round and the primers pAZ102-X and pAZ102-Y in the second round. [14] All samples that resulted positive to the mtLSU-rRNA nested-PCR were subjected to the hemi-nested PCR for DHPS amplification (965 bp fragment length) by the primers PK95 and PK160 in the first round amplification and the primers PK160 and PS876 in the second round. The PCR mixture was used for the mtLSU-rRNA amplification. Negative controls were included in each DNA extraction and amplification round to monitor for possible cross-contamination of samples. A sample was considered positive if the obtained amplification curve signal was below threshold cycle 40. Genotyping for the detection of mutations was based on the sequence analysis of the two loci mtLSU-rRNA and DHPS. The amplified products were purified using the NucleoSpin Extract kit (Macherey-Nagel GmbH & Co. KG, Düren, Germany) and directly sequenced on both strands by Bio-Fab Research s.r.l. (Rome, Italy). Multiple alignments were obtained using ClustalW2 Multiple Sequence Alignments in comparison with other Pneumocystis strains available in GenBank and manually edited with Bioedit software. [15]

The research protocols were performed in concordance with the WMA Helsinki Declaration and its subsequent modification, as well as with Italian National Law n. 675/1996 on the protection of personal data.

### Statistical analysis

The present study provides data on the prevalence of *P. jirovecii* + samples and genotypes, presented on an annual basis as percentage prevalence. The chi-squared test was performed to compare *P. jirovecii* prevalence among different periods under analysis (2014–2016, 2017–2019, 2020–2022). Data that did not follow a normal distribution were expressed as median (interquartile range) and analyzed between groups using the nonparametric Mann-Whitney U test. A two-tailed p-value of less than 0.05 was deemed statistically significant in all analyses. All statistical analyses and graphical representations were performed using SPSS software (version 27; IBM Corp., Armonk, NY, USA).

## Results

The median age of the patients included in the study was 64 years (range: 9–97 years), with a slight male predominance (65,3%, n = 1925). Of note, only six patients aged < 18 years underwent bronchoscopy. Of all 2950 patients tested for molecular detection of *P. jirovecii*, 307 patients (12,1%) tested positive. Specifically, 73/562 (13%) patients tested positive during the 2014–2016 period, 147/921 (16%) during the 2017–2019 period, and 87/1467 (5,9%) during the 2020–2022 pandemic period whit 11 patients presenting Sars-Cov2 and *P. jirovecii* coinfection. Despite a much higher number of samples evaluated, the percentage of positive samples in the 2020–2022 triennium is clearly and significantly lower than in prior periods (p < 0.001). This is different from the previous 2017–2019 period, which showed a linear increase in both the number of samples tested and positive cases from 2014 to 2016.

Overall, the median age of patients who tested positive for *P. jirovecii* was 61 years (range: 20–91 years), the majority of them were male (74%). No differences in gender distribution and median age were observed in the different periods. Infectious Diseases, Hematology, and Pneumology were the most frequent departments of origin of the analyzed samples. During the COVID-19 pandemic we observed an increase in samples from “other departments”, particularly from Emergency Medicine, Intensive Care Unit (ICU) and other “COVID-19” departments (Table 1).

**Table 1:** Department of origin of samples

Department of origin	Period					
	2014–2016		2017–2019		2020–2022	
	n. of samples	PJ+	n. of samples	PJ+	n. of samples	PJ+
Hematology	129 (23%)	12 (16.4%)	199 (22%)	27 (18.4%)	209 (14%)	12 (13.8%)
Infectious Disease	200 (36%)	31 (42.5%)	207 (22.5%)	53 (36.1%)	337 (23%)	26 (30%)
Pneumology	95 (17%)	12 (16.4%)	320 (35%)	30 (20%)	343 (23%)	13 (15%)
Intensive Care Unit	25 (4%)	3 (4%)	15 (2%)	1 (0.7%)	61 (4%)	1 (1.1%)
Other	113 (20%)	15 (20.5%)	180 (29%)	36 (24.5%)	517 (36%)	35 (40%)
Total	562	73	921	147	1467	87

Among the 307 patients positive for *P. jirovecii*, 157 sequences at mtLSU-rRNA locus were successfully obtained. Genotypes were identified on the basis of polymorphisms at nucleotide positions 85 and 248. [16] Four sequence types were isolated: the most frequent (37%) was genotype 1 (85C/248C), followed by genotype 3 (85T/ 248C) and genotype 2 (85A/248C), accounting for 27.4% and 24.8%, while 10.8% of samples showed a mixed genotype, as shown in Table 2. No sample with the sole presence of genotype 4 (85C/248T) was detected.

**Table 2:** Genotypic analysis based on mtSLU-rRNA gene polymorphisms

Genotype	Period		
	2014–2016	2017–2019	2020–2022
1	17 (39.5%)	28 (40.6%)	13 (28.9%)
2	9 (21%)	18 (26.1%)	12 (26.7%)
3	10 (23.3%)	18 (26.1%)	15 (33.3%)
4	0 (0%)	0 (0%)	0 (0%)
Mixed	7 (16.2%)	5 (7.2%)	5 (11.1%)
Total	43	69	45

Notably, the analysis of genotype frequencies shows a trend, although not statistically significant, of a gradual decrease in the number of cases with genotype 1 and an increase in cases with genotype 3 during the study period (Table 2). Genotype 1 is the most frequent in all different groups of patients, while genotype 3 is relatively less common in hematological patients compared to patients admitted in infectious disease and pneumology wards (Table 3).

**Table 3:** Frequencies of genotype based on department of origin

Genotype (total n.)	Department			
	Hematology	Infectious Disease	Pneumology	Other
1 (58)	10 (48%)	19 (32%)	11 (42%)	18 (36%)
2 (39)	5 (24%)	14 (23%)	4 (15%)	16 (32%)
3 (43)	4 (19%)	17 (28%)	9 (35%)	13 (26%)
4 (0)	0	0	0	0
Mixed (17)	2 (9%)	10 (17%)	2 (8%)	3 (6%)
Total	21	60	26	50

## Discussion

In this study, we investigated the frequency of *P. jirovecii* pre and during the COVID-19 pandemic period in our institution. We found that the frequency of patients carrying *P. jirovecii* decreased significantly in the pandemic period compared to the previous era.

The COVID-19 pandemic, caused by the novel coronavirus SARS-CoV-2, has affected millions of people worldwide and has posed unprecedented challenges for the healthcare systems. The diagnosis and management of COVID-19 have been complicated by the possibility of coinfections with other pathogens, including bacteria, fungi, and other viruses. [17–20] Corticosteroids are recommended for treating severe COVID-19 pneumonia; however, caution should be taken due to the risk of developing opportunistic infections due to secondary immunodeficiency in some individuals. [21]

Among the opportunistic coinfections reported in COVID-19 patients, *P.* jirovecii is one of the most concerning, as it shares similar clinical features with COVID-19 (e.g., bilateral multifocal infiltrates and profound hypoxemia) and may worsen the prognosis and increase the mortality. [22–24] Alanio et al. [25] reported a high prevalence (9%) of SARS-Cov2 and *P.Jirovecii* co-occurrence in the ICU setting.However, the incidence and prevalence of PCP in COVID-19 patients are still unknown and may vary depending on the geographic region, the population characteristics, and the diagnostic methods used.

In our study, we used a molecular method (nested-PCR) to detect *P. jirovecii* DNA in BAL samples from patients undergoing bronchoscopy for various indications. We compared the frequency of *P. jirovecii* in three different periods: before (2014–2016, 2017–2019) and during (2020–2022) the emergence of SARS-CoV-2 in our region. We expected to find an increase in *P. jirovecii* frequency in the last period due to the potential coinfection with SARS-CoV-2 and/or the immunosuppressive effect of COVID-19 on the host immune system. However, we found the opposite result: a significant decrease in *P. jirovecii* frequency in the period 2020–2022 compared to the two previous ones. Besides, we observed only 11 patients with simultaneous SARS-CoV-2 and *P.* jirovecii coinfection, of which only one was in the ICU setting. This finding must be interpreted in light of the different politics of management of critically ill patients across the various hospitals and departments. Indeed, at our institution, patients in need of vasopressor and/or invasive ventilation are not always transferred to the ICU but can stay in the hematology/pulmonology or infectious disease departments and be managed in a multidisciplinary fashion with the ICU team. These differences may explain the heterogeneous prevalence in the epidemiology of SARS-Cov2 and *P.jirovecii* observed in ours as opposed to other experiences when looking at data from the individual departments. It is also unclear if SARS-CoV-2 infection should be taken into account as a risk factor for PCP. This is due to the multifactorial nature of immune dysregulation in most patients (HIV, hematological disease, autoimmune disorders, and COVID-19), making any causal link to PCP difficult to establish, as well as any potential bias in the diagnosis of PCP due to clinical features that are shared with COVID-19, which understates the likelihood of actual cases and makes risk factor prediction difficult.

To explain this unexpected finding, we considered several possible factors that could have influenced the epidemiology of *P. jirovecii* during the COVID-19 pandemic. First, one could speculate that the nationwide implementation of NPIs against COVID-19, such as social distancing, wearing masks, and limiting travel, could have reduced the exposure to *P. jirovecii* and consequently decreased the incidence of PCP. This hypothesis is supported by two recent studies from South Korea that reported a similar decrease in PCP frequency in kidney transplant recipients [26] and in patients undergoing bronchoscopy after the introduction of NPIs against COVID-19 [27]. These studies suggested that NPIs could have interrupted or reduced the airborne transmission of *P. jirovecii* among susceptible individuals. Second, we speculated that the changes in the characteristics and management of patients undergoing bronchoscopy during the COVID-19 pandemic could have affected the frequency of *P. jirovecii* detection. For example, we observed a decrease in the number of bronchoscopies performed for suspected lung cancer or interstitial lung disease in 2020–2022 compared to 2017–2019. These conditions are associated with a higher risk of PCP due to underlying immunosuppression or the use of immunosuppressive drugs. However, these factors need to be further investigated with a larger sample size and a more detailed analysis of the patient characteristics and treatments.

Third, the impact of the COVID-19 pandemic on the epidemiology of *P. jirovecii* could have been different in ours compared to other regions of the world. In fact, the incidence and prevalence of *P. jirovecii* vary geographically depending on several factors, such as the prevalence of HIV infection, the availability of prophylaxis and treatment for PCP, the environmental conditions, and the genetic diversity of *P. jirovecii* strains. Moreover, the COVID-19 pandemic has affected different countries and regions with different intensity and timing, depending on the local transmission dynamics, the healthcare system capacity, and the public health response. Therefore, it is possible that the frequency of *P. jirovecii* in COVID-19 patients could be higher or lower in other regions than in ours. A comparison of the risk of PCP in COVID-19 pneumonia between vaccinated and non-vaccinated patients is also the subject of a future study.

Our study has some limitations that should be acknowledged, being the main one its retrospective design that relied on data from electronic medical records and laboratory databases. For instance, data on immunocompetence, comorbidities, and treatments were not available for the majority of patients.

## Conclusion

In conclusion, we found that the frequency of *P. jirovecii* in patients undergoing bronchoscopy decreased significantly in the COVID-19 pandemic era compared to the previous period. We hypothesized that this result could be related to the COVID-19 pandemic and its impact on the epidemiology of *P. jirovecii*, suggesting that the nationwide implementation of NPIs against COVID-19 could have reduced the exposure to *P. jirovecii* and consequently decreased the incidence of PCP. We also discussed other possible factors that could have influenced the frequency of *P. jirovecii* during the COVID-19 pandemic, such as the changes in the characteristics and management of patients undergoing bronchoscopy and the geographic variation of PCP and COVID-19.

The increasing focus on detecting and treating COVID-19 has also resulted in enhanced screening for other infections, such as *P. jirovecii*, another measure further contributing to such findings. Healthcare providers must maintain their vigilance in order to prevent the spread of *P. jirovecii* and other infections, as well as ensuring that patients receive prompt and appropriate treatment for this potentially fatal condition.

## Data Availability

Data supporting this study’s findings are available from the corresponding author upon reasonable request.

## References

[1] Thomas CF, Limper AH (2004). Pneumocystis pneumonia. N Engl J Med.

[2] Catherinot E, Lanternier F, Bougnoux M-E, Lecuit M, Couderc L-J, Lortholary O (2010). Pneumocystis jirovecii Pneumonia. Infect Dis Clin North Am.

[3] Truong J, Ashurst JV. Pneumocystis Jirovecii Pneumonia. StatPearls. StatPearls Publishing; 2022. 29493992

[4] Vera C, Rueda ZV. Transmission and colonization of Pneumocystis jirovecii. J Fungi Basel Switz 2021, 7. 10.3390/jof7110979 PMC8622989 34829266

[5] Valade S, Damiani C, Derouin F, Totet A. Menotti: Pneumocystis jirovecii airborne transmission between critically ill patients and health care workers. Intensive Care Med 2015, 41. 10.1007/s00134-015-3835-9 25940964

[6] Fujii T, Nakamura T, Iwamoto A (2007). Pneumocystis pneumonia in patients with HIV infection: clinical manifestations, laboratory findings, and radiological features. J Infect Chemother off J Jpn Soc Chemother.

[7] Barbounis V, Aperis G, Gambletsas E, Koumakis G, Demiris M, Vassilomanolakis M, Efremidis A. Pneumocystis Carinii Pneumonia in patients with solid tumors and lymphomas: predisposing factors and outcome. ANTICANCER Res 2005. 15816641

[8] Roux A, Canet E, Valade S, Gangneux-Robert F, Hamane S, Lafabrie A, Maubon D, Debourgogne A, Le Gal S, Dalle F (2014). *Pneumocystis jirovecii* Pneumonia in patients with or without AIDS, France. Emerg Infect Dis.

[9] Paterno G, Guarnera L, Palmieri R, Del Prete V, Bonanni F, Buzzatti E, Moretti F, Casciani P, Savi A, Di Cave D (2022). *Pneumocystis jirovecii* pneumonia in patients with previously untreated acute myeloid leukaemia. Mycoses.

[10] WHO Director-. General’s opening remarks at the media briefing on COVID-19–11 March 2020. [date unknown].

[11] Perra N (2021). Non-pharmaceutical interventions during the COVID-19 pandemic: a review. Phys Rep.

[12] Bateman M, Oladele R, Kolls JK (2020). Diagnosing pneumocystis jirovecii pneumonia: a review of current methods and novel approaches. Med Mycol.

[13] Sasso M, Chastang-Dumas E, Bastide S, Alonso S, Lechiche C, Bourgeois N, Lachaud L (2016). Performances of Four Real-Time PCR assays for diagnosis of Pneumocystis jirovecii Pneumonia. J Clin Microbiol.

[14] Alanio A, Gits-Muselli M, Guigue N, Desnos-Ollivier M, Calderon EJ, Di Cave D, Dupont D, Hamprecht A, Hauser PM, Helweg-Larsen J (2017). Diversity of Pneumocystis jirovecii Across Europe: a Multicentre Observational Study. EBioMedicine.

[15] Dimonte S, Berrilli F, D’Orazi C, D’Alfonso R, Placco F, Bordi E, Perno CF, Di Cave D (2013). Molecular analysis based on mtLSU-rRNA and DHPS sequences of Pneumocystis jirovecii from immunocompromised and immunocompetent patients in Italy. Infect Genet Evol.

[16] Beard CB, Carter JL, Keely SP, Huang L, Pieniazek NJ, Moura IN, Roberts JM, Hightower AW, Bens MS, Freeman AR (2000). Genetic variation in Pneumocystis carinii isolates from different geographic regions: implications for transmission. Emerg Infect Dis.

[17] Swets MC, Russell CD, Harrison EM, Docherty AB, Lone N, Girvan M, Hardwick HE, Investigators ISARIC4C, Visser LG, Openshaw PJM (2022). SARS-CoV-2 co-infection with influenza viruses, respiratory syncytial virus, or adenoviruses. Lancet Lond Engl.

[18] Mina S, Yaakoub H, Annweiler C, Dubée V, Papon N (2022). COVID-19 and fungal infections: a double debacle. Microbes Infect.

[19] Moreno-García E, Puerta-Alcalde P, Letona L, Meira F, Dueñas G, Chumbita M, Garcia-Pouton N, Monzó P, Lopera C, Serra L (2022). Bacterial co-infection at hospital admission in patients with COVID-19. Int J Infect Dis IJID off Publ Int Soc Infect Dis.

[20] Ergün M, Brüggemann RJM, Alanio A, Dellière S, van Arkel A, Bentvelsen RG, van Rijpstra T, der Brugge S, Lagrou K, Janssen NAF et al. Aspergillus Test Profiles and Mortality in critically ill COVID-19 patients. J Clin Microbiol [date unknown], 59:e01229–21. 10.1128/JCM.01229-21 PMC8601217 34495710

[21] Horby P, Lim WS, Emberson JR, Mafham M, Bell JL, Linsell L, Staplin N, Brightling C, Ustianowski A, RECOVERY Collaborative Group (2021). Dexamethasone in hospitalized patients with Covid-19. N Engl J Med.

[22] Gioia F, Albasata H, Hosseini-Moghaddam SM (2022). Concurrent infection with SARS-CoV-2 and Pneumocystis jirovecii in Immunocompromised and Immunocompetent individuals. J Fungi Basel Switz.

[23] Raffaelli F, Tanzarella ES, De Pascale G, Tumbarello M (2022). Invasive respiratory fungal infections in COVID-19 critically ill patients. J Fungi Basel Switz.

[24] Takahashi T, Saito A, Kuronuma K, Nishikiori H, Chiba H (2022). Pneumocystis jirovecii Pneumonia Associated with COVID-19 in patients with interstitial pneumonia. Med Kaunas Lith.

[25] Alanio A, Dellière S, Voicu S, Bretagne S, Mégarbane B (2021). The presence of Pneumocystis jirovecii in critically ill patients with COVID-19. J Infect.

[26] Park KH, Jung C-Y, Jeong W, Lee G, Yang JS, Nam CM, Kim HW, Kim BS (2022). Nationwide implementation of nonpharmaceutical interventions during the Coronavirus Disease 2019 pandemic is Associated with decreased incidence of pneumocystis jirovecii pneumonia in kidney transplant recipients. Open Forum Infect Dis.

[27] Kim D, Kim SB, Jeon S, Kim S, Lee KH, Lee HS, Han SH (2021). No change of Pneumocystis jirovecii Pneumonia after the COVID-19 pandemic: Multicenter Time-Series analyses. J Fungi Basel Switz.

